# Presence of mechanical dyssynchrony in Duchenne Muscular dystrophy: a cardiac MRI study utilizing cross correlation delay

**DOI:** 10.1186/1532-429X-13-S1-O17

**Published:** 2011-02-02

**Authors:** Kan N Hor, Janaka P Wansapura, Hussein R Al-Khalidi, William M Gottliebson, Michael D Taylor, Richard JCzosek, Sherif F Nagueh, Nandakishore Akula, Eugene S Chung, D Woodrow Benson, Wojciech Mazur

**Affiliations:** 1 CCHMC, Cincinnati, OH, USA; 2 Duke University School of Medicine, Durham, NC, USA; 3 Methodist Hospital, Houston, TX, USA; 4 The Heart and Vascular Center at The Christ Hospital, Cincinnati, OH, USA

## Introduction

Cardiac dysfunction in boys with Duchenne muscular dystrophy (DMD) is a leading cause of death. Cardiac resynchronization therapy (CRT) has been shown to dramatically decrease mortality in eligible adult population with congestive heart failure. We hypothesized that mechanical dyssynchrony is present in DMD patients and that cardiac magnetic resonance imaging (CMR) may predict CRT efficacy**.**

## Purpose

We hypothesized that mechanical dyssynchrony is present in DMD patients and that cardiac magnetic resonance imaging (CMR) may predict CRT efficacy.

## Methods

**Figure 1:**
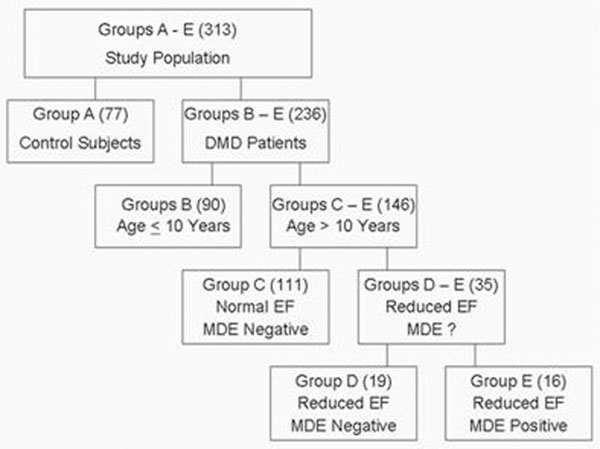


DMD patients (n=236) were stratified into 4 groups (B-D, Figure 1) based on age, left ventricular (LV) ejection fraction (EF) and presence of myocardial fibrosis defined as positive myocardial delayed enhancement (MDE) compared to normal controls (group A, n=77). Dyssynchrony indices were calculated based on timing of CMR derived circumferential strain (ε_cc_). The calculated indices included cross-correlation delay (XCD), uniformity of strain (US), regional vector of variance (RVV), time to maximum strain (TTMS) and standard deviation (SD) of TTMS. Abnormal XCD value was defined as > normal + 2SD. US, RVV, TTMS and SD were than derived for all patient population and patient with dyssynchrony defined as abnormal XCD.

## Results

There was overall low prevalence of circumferential dyssynchrony in the entire DMD population (3%); it increased to 17.1% for patients with abnormal EF and to 31.2% in the most advanced stage (abnormal EF with fibrosis) (Table 1 and 2). All but one DMD patient with mechanical dyssynchrony exhibited normal QRS duration suggesting absence of electrical dyssynchrony. The calculated US and RVV values (0.91 ± 0.09, 1.34 ± 0.48) indicate disperse rather than clustered dyssynchrony.

**Table 1:** General Characteristic by Group

	Group				
Parameter	A (n=77)	B (n=90)	C (n=111)	D (n=19)	E (n=16)

Age (years) (min, max)	13.9 ± 8.9 (4.8, 46.6)	8.5 ± 0.9** (6.6, 10.0)	12.9 ± 2.8 (10.0, 24.8)	15.0 ± 3.9 (10.3, 24.0)	17.3 ± 5.4** (8.7, 26.4)
LVEF (%) (min, max)	64.6 ± 5.9 (48.9, 76.2)	65.0 ± 4.8 (55.2, 82.7)	64.4 ± 5.8 (55.3, 83.3)	49.4 ± 6.6** (31.6, 54.4)	36.5 ± 12.2** (17.3, 54.0)
XCD^a^(ms)	45.6 ± 16.9 (0, 83.3)	35.3 ± 16.6 (0, 70.5)	38.2 ± 19.8 (0, 112.5)	52.2 ± 22.4 (27.5, 105)	73.0 ± 41.9 (21.3, 157.5)
US_max (min, max)	0.97 ± 0.04 (0.84, 1.00)	0.98 ± 0.03 (0.90, 1.00)	0.97 ± 0.03 (0.86, 1.00)	0.97 ± 0.04 (0.88, 1.00)	0.92 ± 0.08** (0.73, 1.00)
TTMS (min, max)	78.2 ± 31.8 (0.0, 148.8)	65.4 ± 22.9** (21.3, 157.5)	71.2 ± 24.0 (21.3, 105.8)	95.1 ± 19.6** (56.5, 140.0)	111.4 ± 33.0** (63.8, 157.5)
RVV_max (min, max)	0.95 ± 0.57 (0.10, 2.8)	1.03 ± 0.55 (0.04, 2.7)	1.13 ± 0.57 (0.14, 2.7)	1.03 ± 0.51 (0.35, 2.16)	1.34 ± 0.57** (0.42, 2.2)
STD_peak (min, max)	31.7 ± 12.0 (0, 56.1)	26.7 ± 8.4** (11.6, 55.1)	29.6 ± 9.7 (8.7, 52.7)	36.5 ± 6.9 (21.3, 47.8)	46.1 ± 16.6** (24.8, 75.7)
QRS (min, max)	92.8 ± 11.41	85.1 ± 7.8** (70, 104)	86.6 ± 8.0** (68, 109)	86.1 ± 13.4 (68, 126)	97.1 ± 21.9 (80, 134)
ε_cc_ (min, max)	-18.2 ± 4.5 (-17.4, -25.5)	-14.2 ± 1.4** (-10.4, -16.3)	-13.2 ± 2.0** (-6.5, -16.5)	-10.7 ± 2.1 (-7.4, -14.2)	-7.0 ± 2.8 (-2.8, -11.9)

ε_cc_ = Circumferential strain, Group A = Control subjects, Group B = DMD age ≤10 yrs, Group C = DMD age > 10 yrs with normal EF and MDE negative, Group D = DMD age > 10 yrs with reduced EF and MDE negative, Group E = DMD age > 10 years with reduced EF and positive MDE, LVEF = Left ventricular ejection fraction, MDE = myocardial delayed enhancement, STD = Standard deviation, TTMS = Time to maximum strain, US = Uniformity of strain, RVV = Regional vector of variance, XCD = Cross correlation delay, XCD^a^ = maximum of (XCD 1-4, XCD 2-5 and XCD 3.6).

**Table 2:** Abnormal XCDs vs. the control

	Group	
Parmaeter	Control (n=77)	Abnormal XCDs (n=9)

Age (min, max)	13.9 ± 8.9 (4.8, 46.6)	16.7 ± 5.0 (10.9, 24.3)
LVEF (%) (min, max)	64.6 ± 5.9 (48.9, 76.2)	43.9 ± 16.7** (17.3, 61.9)
XCD^a^ (min, max)	45.6 ± 16.9 (0, 83.3)	116.5 ± 20.5** (93.8, 157.5)
Presence of MDE	0 (0.0%)	4 (57.1%)**
US_max (min, max)	0.97 ± 0.04 (0.84, 1.00)	0.91 ± 0.09** (0.73, 0.98)
TTMS (min, max)	78.2 ± 31.8 (0.0, 148.8)	111.2 ± 39.5** (37.5, 157.5)
RVV_max (min, max)	0.95 ± 0.57 (0.10, 2.8)	1.34 ± 0.48 (0.42, 2.0)
STD_peak (min, max)	31.7 ± 12.0 (0, 56.1)	47.6 ± 18.1**
QRS (min, max)	92.8 ± 11.4^1^ (74, 122)	98.2 ± 15.3 (86, 134)

## Conclusion

Mechanical dyssynchrony is frequent in boys with end stage DMD-associated cardiac dysfunction. It is associated with normal QRS complex as well as extensive lateral fibrosis. Based on these findings, it is unlikely that this patient population will benefit from CRT.

